# Supplementary material for: Difficulties in Accessing Cancer Care in a Small Island State: A Community-Based Pilot Study of Cancer Survivors in Saint Lucia
Source: Int J Environ Res Public Health. 2021 Apr 29;18(9):4770. doi: 10.3390/ijerph18094770 (PMC8124473; doi:10.3390/ijerph18094770)
Supplement: Supplementary file 1 [file ijerph-18-04770-s001.zip › Supplementary files IJERPH_Cancer SLU/Questionnaire DCAP_patients_final (Supp Material).pdf]

Vaughan A. Lewis Institute for Research and Innovation

Health services research

Description of Cancer Health Services: Diagnosis and Treatment Pathways

DCAP Study (Saint Lucia)

Patient interview questionnaire

Patient study ID number:

758- 0 |__|__| - |__ | __| __|

Supplementary materials

I. Information relative to the interview

1. Interview number ……………………………
2. Date of interview |__|__|/|__|__|/|__|__| (dd/mm/yy)
3. Time of interview |__|__|h |__|__|
4. Patient study ID number: 758 – 0 |__|__| - |__ | __| __|

II. Socio-demographics

Note to interviewer: prompt for beginning of interview: "This interview is separated into two main parts. Firstly, I will ask you some question about yourself and your living conditions and secondly we will talk about your experience in accessing care in Saint Lucia. If you do not understand something or if you have any questions, feel free to interrupt me."

1. Sex of patient ☐1 Male ☐2 Female
2. Ethnicity: ☐1 Black ☐2 White ☐3 Other
3. Date of birth |__|__|/|__|__|/|__|__| (dd/mm/yy)
4. Place of residence
   a. Street address or neighbourhood: ……………………………………
   b. District/Parish: ………………………………

5. Cancer diagnosis:

Date first diagnosed |__|__|/|__|__|/|__|__| (dd/mm/yy)

6. Type of cancer ……………………………………………… (breast, colon, lung etc)
7. Stage of cancer: ☐1 I ☐2 II ☐3 III ☐4 IV ☐5 I don't know

8. Marital Status

☐1 Single
☐2 Married
☐3 Divorced or separated
☐4 Widowed

9. What is the highest level of formal education you have completed?

☐1 Primary school
☐2 Secondary school (CXC/CSEC or O level)
☐3 Community college (Advanced level certificate, Associates degree)
☐4 University or Higher learning degree
☐5 Other ……………………

Supplementary materials

10. Do you work currently (i.e. professional activity generating revenue)?

☐1 Yes ☐2 No, Stopped working

11. If no, what are the circumstances of you ending your professional activity

☐1 Retirement
☐2 Unemployment
☐3 Invalidity or for sickness
☐4 Other reason……………………….…

12. In What year did you stop working? ……………………

13. Are you covered by private health insurance policy?

☐1 Yes ☐2 No

14. Are you the one paying for it?

☐1 Yes ☐2 No

15. Do you have a water heating system at your home?

☐1 Yes ☐2 No ☐3 I don't know

16. How would you best describe your water heating installation?

☐1 Electrical ☐2 Solar ☐3 Other _____________ ☐4 I don't know

17. Have you been treated before for any of the conditions below or is there any medical condition you have been suffering from **prior** to your diagnosis? (tick 'yes' or 'no' for each condition):

Condition	Response		
Heart disease	☐1 Yes	☐2 No	☐3 Don't know
Stroke	☐1 Yes	☐2 No	☐3 Don't know
Lung disease	☐1 Yes	☐2 No	☐3 Don't know
Diabetes	☐1 Yes	☐2 No	☐3 Don't know
Other:			

Supplementary materials

III. First presentation/clinical appearance

Note to interviewer: prompt for beginning of section IV: "I am going to ask you some questions about what happened before you were diagnosed and the different health care provider that you saw.

1. Did you notice any change in your body or manifestation of symptoms prior to seeing a health care provider? (e.g. Lump in breast, sore throat, blood in stool)

   ☐$_1$ Yes ☐$_2$ No

   ………

   ………

   ………

   Note to interviewer: If "No" for question 1, skip to question 6: "Was your cancer diagnosis a result of etc".

2. Do you recall **when you first noticed** this physical change in your body?

   |__|__|/|__|__|/|__|__| (dd/mm/yy)

3. Can you list the symptoms or body changes that you experienced? (e.g. Swelling in breast or armpit, Fatigue, Unexplained weight loss, Loss of appetite)

   Symptom 1: ………………………………………………………………………………………………

   Symptom 2: ………………………………………………………………………………………………

   Symptom 3: ………………………………………………………………………………………………

4. Following the appearance of these body changes, what was your reaction?

   ☐$_1$ I contacted a health care professional (HCP)
   ☐$_2$ I monitored the body changes /symptoms passively
   ☐$_3$ I ignored the body changes /symptoms
   ☐$_4$ Other (specify below):

   ………

   ………

   ………

Supplementary materials

5. What compelled you to seek help from a health care professional (HCP) following the appearance of the body changes (or symptoms).

☐1 The body changes/symptoms became very apparent

☐2 I had a gut feeling something was wrong

☐3 Was encouraged by close friends/family to go to a HCP

☐4 Other (specify below):

....

....

....

6. Was your cancer diagnosis a result of an investigation by health care professional for another health concern during which time the cancer was discovered (e.g. a cancer **screening test**? (mammography, colonoscopy etc))

☐1 Yes ☐2 No

If no, and no body changes were noticed specify the circumstances surrounding the discovery of the tumour:

....

....

....

Supplementary materials

Consultation with a professional - N°1

1. Who was the **first** health care professional you consulted about your symptoms? Including General practitioner, Specialist doctor and Naturopathic professionals.

Note to interviewer: If "Yes" for section III, question 6 (cancer diagnosis a result of an investigation…etc.) (Case of screening) ask instead for the "first HCP you consulted when your cancer was discovered",

☐1 General practitioner (GP)

☐2 Specialist doctor (gynaecologist etc.) ……………………………………………….

☐3 Herbalist

☐4 Naturopathic professional

☐5 Other allied health professional : ……………………………………………….

☐6 Other ……………………………………………….

Establishment: ………………………………………… Country; ……………………………………………

………..

……

2. Did you contact this health care provider to schedule an appointment prior to seeing him/her for a consultation?

☐1 Yes ☐2 No

Note to interviewer: If No, i.e. the patient did not schedule an appointment ahead of the consultation with this health care provider, skip to question 5 "Do you recall what date you had that consultation".

3. Do you recall what date **an appointment was made** with this health care provider?

|__|__|/|__|__|/|__|__| (dd/mm/yy).

Supplementary materials

Note to interviewer: If the patient cannot recall the exact date, fill in the table below in Q4.

4. How long did you wait before making an appointment with this health care provider after having your health concern(s) or symptom(s)? Please tick only one answer.

   Please tick only one answer:

☐1 Same day/Next day (<24h)	☐2 Days	☐3 Weeks	☐3 Months	☐4 Cannot recall
	Specify the number of **days**	Specify the number of **weeks**	Specify the number of **months**	
	|__||__|	|__||__|	|__||__|	

Note to interviewer: If a precise time interval cannot be given in months, weeks or days try to suggest the following ranges. (DO NOT fill out the table and select a range)

☐1 Less than 1 week
☐2 1 to 2 weeks
☐3 3 to 4 weeks
☐4 5 to 7 weeks
☐5 2 to 5 months
☐6 6 to 12 months
☐7 Greater than 12 months

5. Do you recall what date you had that **consultation** with a health care provider? |__|__|/|__|__|/|__|__| (dd/mm/yy).

Note to interviewer: If the patient cannot recall the exact date, fill in the table below in Q6.

Supplementary materials

6. Approximately how long did it take to **see this health care provider for a consultation:**

☐1 After having your health concern(s) or symptom(s)? (Tick if **no appointment** was scheduled)

☐2 After contacting them for an appointment (Tick if an **appointment** was scheduled)

Please tick only one answer:

<table>
<tr><td rowspan="3">☐1Same day/Next day (<24h)</td><td>☐2Days</td><td>☐3Weeks</td><td>☐3Months</td><td rowspan="3">☐4Cannot recall</td></tr>
<tr><td>Specify the number of days</td><td>Specify the number of weeks</td><td>Specify the number of months</td></tr>
<tr><td>|__||__|</td><td>|__||__|</td><td>|__||__|</td></tr>
</table>

Note to interviewer: If a precise time interval cannot be given in months, weeks or days try to suggest the following ranges. (DO NOT fill out the table and select a range)

☐1 Less than 1 week

☐2 1 to 2 weeks

☐3 3 to 4 weeks

☐4 5 to 7 weeks

☐5 2 to 5 months

☐6 6 to 12 months

☐7 Greater than 12 months

7. Can you list the symptoms or body changes that you were experiencing at the moment of the consultation with this health care provider?

Symptom 1: ………………………………………………………………………………………… ……………

Symptom 2: ………………………………………………………………………………………… …………….

Symptom 3: ………………………………………………………………………………………… ……………

Supplementary materials

8. Could you explain what happened during that consultation?

……

……

……

……

……

9. Were any diagnostic tests/treatments **prescribed** at the initial visit?

Diagnosis test: ☐1 Yes ☐2 No

Please list tests, e.g. chest x-ray, CT, Mammography

Test 1 ……………………………………………

Test 2 ……………………………………………

Test 3 ……………………………………………

Please list Treatment, e.g. hormone therapy, nutrient supplement

Treatment: ☐1 Yes ☐2 No

Treatment 1 ……………………………………………

Treatment 2 ……………………………………………

Treatment 3 ……………………………………………

……

……

……

10. Were you referred to anyone during your visit with the health care provider

☐1 Yes ☐2 No

Supplementary materials

11. If yes, to who were you referred?

☐1 General practitioner (GP)
☐2 Specialist Doctor
☐3 Naturopathic professional
☐4 Herbalist
☐5 Hospital
☐6 Hospital, emergency admission
☐7 Other :

……………………………………

Speciality ……………………. Health care provider name ……………………….

Hospital /establishment name ………………………………….

Country …………………….………….

………

………..

12. Did you schedule a review appointment with this health care provider?

☐1 Yes ☐2 No, nothing further

13. What was the review period for this review appointment with this health care provider?

☐1 Less than 1 week
☐2 1 to 2 weeks
☐3 3 to 4 weeks
☐4 Greater than 4 weeks

14. Can you suggest any improvements? (in regards to the care from this professional)

………

………..

………

15. What happened after this initial appointment?

………

………..

………

………..

Supplementary materials

Consultation with a professional - N°2

1. What health care professional did you consult following the last one?

☐1 General practitioner (GP)

☐2 Specialist doctor (gynaecologist etc.) ………………………………………………..

☐3 Herbalist

☐4 Naturopathic professional

☐5 Other allied health professional: ………………………………………………..

☐6 Other ………………………………………………..

Establishment: ………………………………………… Country; ……………………………………………

…….

…….

2. Did you contact this health care provider to schedule an appointment prior to seeing him/her for a consultation?

☐1 Yes ☐2 No

Note to interviewer: If No, i.e. the patient did not schedule an appointment ahead of the consultation with this health care provider, skip to question 5 “Do you recall what date you had that consultation”.

3. Do you recall what date **an appointment was made** with this health care provider?

|__|__|/|__|__|/|__|__| (dd/mm/yy).

Supplementary materials

Note to interviewer: If the patient cannot recall the exact date, fill in the table below in Q4.

4. How long did you wait before making an appointment with this health care provider after having your health concern(s) or symptom(s)? Please tick only one answer.

   ☐$_1$ After having consulted the last Health care provider?

   ☐$_2$ After having your health concern(s) or symptom(s)?

   Please tick only one answer:

☐$_1$Same day/Next day (<24h)	☐$_2$Days	☐$_3$Weeks	☐$_3$Months	☐$_4$Cannot recall
	Specify the number of **days**	Specify the number of **weeks**	Specify the number of **months**	
	|__||__|	|__||__|	|__||__|	

Note to interviewer: If a precise time interval cannot be given in months, weeks or days try to suggest the following ranges. (DO NOT fill out the table and select a range)

☐$_1$ Less than 1 week

☐$_2$ 1 to 2 weeks

☐$_3$ 3 to 4 weeks

☐$_4$ 5 to 7 weeks

☐$_5$ 2 to 5 months

☐$_6$ 6 to 12 months

☐$_7$ Greater than 12 months

5. Do you recall what date you had that **consultation** with a health care provider? If you cannot remember the exact date, you can provide the month and the year.

   |__|__|/|__|__|/|__|__| (dd/mm/yy).

Note to interviewer: If the patient cannot recall the exact date, fill in the table below in Q6.

Supplementary materials

6. Approximately how long did it take to **see this health care provider for a consultation:**

(Tick only if **no appointment** was scheduled: walk-in visit)

☐1 After having consulted the last Health care provider?

☐3 After having your health concern(s) or symptom(s)?

(Tick only if an **appointment** was scheduled)

☐3 After contacting them for an appointment

Please tick only one answer:

☐1Same day/Next day (<24h)	☐2Days	☐3Weeks	☐3Months	☐4Cannot recall
	Specify the number of **days**	Specify the number of **weeks**	Specify the number of **months**	
	|__||__|	|__||__|	|__||__|	

Note to interviewer: If a precise time interval cannot be given in months, weeks or days try to suggest the following ranges. (DO NOT fill out the table and select a range)

☐1 Less than 1 week

☐2 1 to 2 weeks

☐3 3 to 4 weeks

☐4 5 to 7 weeks

☐5 2 to 5 months

☐6 6 to 12 months

☐7 Greater than 12 months

7. Can you list the symptoms or body changes that you were experiencing at the moment of the consultation with this health care provider?

Symptom 1: ……………………………………………………………………………………………………

Symptom 2: ……………………………………………………………………………………………………

Symptom 3: ……………………………………………………………………………………………………

Supplementary materials

8. Could you explain what happened during that consultation?

……

……

……

……

……

9. Were any diagnostic tests/treatments **prescribed** at the initial visit?

Diagnosis test: ☐$_1$ Yes ☐$_2$ No

Please list tests, e.g. chest x-ray, CT, Mammography

Test 1 ……………………………………………

Test 2 ……………………………………………

Test 3 ……………………………………………

Please list Treatment, e.g. hormone therapy, nutrient supplement

Treatment: ☐$_1$ Yes ☐$_2$ No

Treatment 1 ……………………………………………

Treatment 2 ……………………………………………

Treatment 3 ……………………………………………

……

……

……

10. Were you referred to anyone during your visit with the health care provider

☐$_1$ Yes ☐$_2$ No

Supplementary materials

11. If yes, to who were you referred?

☐1 General practitioner (GP)
☐2 Specialist Doctor
☐3 Naturopathic professional
☐4 Herbalist
☐5 Hospital
☐6 Hospital, emergency admission
☐7 Other :

……………………………………

Speciality …………………… Health care provider name ……………………….

Hospital /establishment name ………………………………….

Country ……………………………….

……

……

12. Did you schedule a review appointment with this health care provider?

☐1 Yes ☐2 No, nothing further

13. What was the review period for this review appointment with this health care provider?

☐1 Less than 1 week
☐2 1 to 2 weeks
☐3 3 to 4 weeks
☐4 Greater than 4 weeks

14. Can you suggest any improvements? (in regards to the care from this professional)

……

……

……

15. What happened after this initial appointment?

……

……

……

……

Supplementary materials

Consultation with a professional - N°3

16. What health care professional did you consult following the last one?

☐1 General practitioner (GP)

☐2 Specialist doctor (gynaecologist etc.) ………………………………………………..

☐3 Herbalist

☐4 Naturopathic professional

☐5 Other allied health professional : ………………………………………………..

☐6 Other ………………………………………………..

Establishment: ………………………………………   Country; …………………………………………

…….

…….

17. Did you contact this health care provider to schedule an appointment prior to seeing him/her for a consultation?

☐1 Yes   ☐2 No

Note to interviewer: If No, i.e. the patient did not schedule an appointment ahead of the consultation with this health care provider, skip to question 5 "Do you recall what date you had that consultation".

18. Do you recall what date an appointment was made with this health care provider?

|__|__|/|__|__|/|__|__| (dd/mm/yy).

Supplementary materials

Note to interviewer: If the patient cannot recall the exact date, fill in the table below in Q4.

19. How long did you wait before making an appointment with this health care provider after having your health concern(s) or symptom(s)? Please tick only one answer.

☐1 After having consulted the last Health care provider?

☐2 After having your health concern(s) or symptom(s)?

Please tick only one answer:

☐1 Same day/Next day (<24h)	☐2 Days	☐3 Weeks	☐3 Months	☐4 Cannot recall
	Specify the number of **days**	Specify the number of **weeks**	Specify the number of **months**	
	|__||__|	|__||__|	|__||__|	

Note to interviewer: If a precise time interval cannot be given in months, weeks or days try to suggest the following ranges. (DO NOT fill out the table and select a range)

☐1 Less than 1 week

☐2 1 to 2 weeks

☐3 3 to 4 weeks

☐4 5 to 7 weeks

☐5 2 to 5 months

☐6 6 to 12 months

☐7 Greater than 12 months

20. Do you recall what date you had that **consultation** with a health care provider? If you cannot remember the exact date, you can provide the month and the year.

|__|__|/|__|__|/|__|__| (dd/mm/yy).

Note to interviewer: If the patient cannot recall the exact date, fill in the table below in Q6.

Supplementary materials

21. Approximately how long did it take to **see this health care provider for a consultation:**

(Tick only if **no appointment** was scheduled: walk-in visit)

☐1 After having consulted the last Health care provider?

☐3 After having your health concern(s) or symptom(s)?

(Tick only if an **appointment** was scheduled)

☐3 After contacting them for an appointment

Please tick only one answer:

☐1Same day/Next day (<24h)	☐2Days	☐3Weeks	☐3Months	☐4Cannot recall
	Specify the number of **days**	Specify the number of **weeks**	Specify the number of **months**	
	|__||__|	|__||__|	|__||__|	

Note to interviewer: If a precise time interval cannot be given in months, weeks or days try to suggest the following ranges. (DO NOT fill out the table and select a range)

☐1 Less than 1 week

☐2 1 to 2 weeks

☐3 3 to 4 weeks

☐4 5 to 7 weeks

☐5 2 to 5 months

☐6 6 to 12 months

☐7 Greater than 12 months

22. Can you list the symptoms or body changes that you were experiencing at the moment of the consultation with this health care provider?

Symptom 1: ……………………………………………………………………………………………………

Symptom 2: ……………………………………………………………………………………………………

Symptom 3: ……………………………………………………………………………………………………

Supplementary materials

23. Could you explain what happened during that consultation?

……

……

……

……

……

24. Were any diagnostic tests/treatments **prescribed** at the initial visit?

Diagnosis test: ☐1 Yes ☐2 No

Please list tests, e.g. chest x-ray, CT, Mammography

Test 1 ………………………………………….

Test 2 ………………………………………….

Test 3 ………………………………………….

Please list Treatment, e.g. hormone therapy, nutrient supplement

Treatment: ☐1 Yes ☐2 No

Treatment 1 ………………………………………….

Treatment 2 ………………………………………….

Treatment 3 ………………………………………….

……

……

……

25. Were you referred to anyone during your visit with the health care provider

☐1 Yes ☐2 No

Supplementary materials

26. If yes, to who were you referred?

☐1 General practitioner (GP)
☐2 Specialist Doctor
☐3 Naturopathic professional
☐4 Herbalist
☐5 Hospital
☐6 Hospital, emergency admission
☐7 Other :

……………………………………

Speciality …………………… Health care provider name ……………………….

Hospital /establishment name ………………………………….

Country ……………………………….

………

………..

27. Did you schedule a review appointment with this health care provider?

☐1 Yes ☐2 No, nothing further

28. What was the review period for this review appointment with this health care provider?

☐1 Less than 1 week
☐2 1 to 2 weeks
☐3 3 to 4 weeks
☐4 Greater than 4 weeks

29. Can you suggest any improvements? (in regards to the care from this professional)

………

………..

………

30. What happened after this initial appointment?

………

………..

………

………..

Supplementary materials

V. First investigations

1. Following your visit(s) with your health care provider(s) did you have any diagnostic tests done?

□1 Yes □2 No

2. Could you please list the all tests that were done? Where possible provide the place and date of the test as well as the date the result was issued (Fill out the table below for the diagnosis test **performed**)

Test performed (Blood test, Chest X-ray etc)	Lab /location of test	Date of test (dd/mm/yy)	Time taken for results to be issued			Suspicion of cancer
			Same day	Number of **days**	Number of **weeks**	
			□			□1Yes □2No
			□			□1Yes □2No
			□			□1Yes □2No
			□			□1Yes □2No

....

....

....

Supplementary materials

Note to interviewer: If the length of time was more than 2 weeks, ask Question 3 “What were the reasons for your delay. Otherwise, skip to question 5 “What happened next”

3. According to you, what was/were the reasons contributing to this delay in the issuance of diagnosis test results

………

………

………

4. Can you suggest any improvements to this process?

………

………

………

5. What happened next? (After final diagnosis test(s) was(were) performed)

………

………

………

Supplementary materials

VI. Diagnosis announcement

1. What was the date you were **told** you had cancer? If you cannot remember the exact date, you can fill in the month and the year

   |__|__|/|__|__|/|__|__| (dd/mm/yy).

Note to interviewer: If the person cannot remember the date from the previous questions skip to question 2 "Who made the announcement of your cancer diagnosis to you?".

2. Who made the announcement of your cancer diagnosis to you? (e.g. GP, Surgeon, Respiratory physician)

☐1 General practitioner (GP)
☐2 Specialist (gynaecologist etc.)
☐3 Nurse
☐4Other ………………………………….

3. Was the explanation and information provided clear and adequate?

☐1- Very unclear/inadequate
☐2- Unclear/inadequate
☐3-Acceptable
☐4- Clear/adequate
☐5- Very clear/adequate

………

………

4. How could it have been done better?

………

………

………

5. What happened following the announcement of your diagnosis?

………

………

………

………

………

Supplementary materials

VII. Cancer treatment

Note to interviewer: Beginning of treatment is defined as the moment that a treatment prescribed by a HCP is administered to a patient for the first time regardless of the treatment modality.

1. Have you initiated any treatment for the time being (surgery/ chemotherapy/ radiotherapy/ alternative medicine etc.)?

   ☐$_1$ Yes ☐$_2$ No

2. What treatment modality did you start with first? (Including alternative medicine)

   ☐$_1$ Surgery ☐$_2$ Chemotherapy ☐$_3$ Other ……………………………….

3. What was the date you began that first treatment for your cancer?

   |__|__|/|__|__|/|__|__| (dd/mm/yy).

   Note to interviewer: If the patient cannot recall the exact date, fill in the table below in Q4.

4. How long was it from when you were diagnosed to when you started treatment (first surgery, chemotherapy etc)?

☐$_1$Same day/Next day (<24h)	☐$_2$Days	☐$_3$Weeks	☐$_3$Months	☐$_4$Cannot recall
	Specify the number of **days**	Specify the number of **weeks**	Specify the number of **months**	
	|__||__|	|__||__|	|__||__|	

Note to interviewer: If a precise time interval cannot be given in months, weeks or days try to suggest the following ranges. (DO NOT fill out the table and select a range)

☐$_1$ Less than 1 week

☐$_2$ 1 to 2 weeks

☐$_3$ 3 to 4 weeks

☐$_4$ 5 to 7 weeks

☐$_5$ 2 to 5 months

☐$_6$ 6 to 12 months

☐$_7$ Greater than 12 months

……

……

Supplementary materials

Note to interviewer: If the length of time was less than a week, skip to Q6 “Did you have an opportunity to discuss your treatment options with someone?”

5. According to you, what was/were the reasons contributing to this delay in treatment (multiple answers are possible)

☐ Personal decision to wait
☐ Hospital waiting times
☐ Cancelled or postponed appointment
☐ I don’t know
☐ Other: …………………………..

………

………

………

6. Did you have an opportunity to discuss your treatment options with someone (friends/family/colleagues) prior to initiating treatment?

☐1 Yes ☐2 No ☐3 I don’t know

………

………

Supplementary materials

7. I will give you a list of cancer treatment modalities and I would like you to tell me if you had that treatment or not. If so, please can you estimate the date this treatment started? Please tick all that apply.

Type of Treatment			Treatment date (dd/mm/yy)	Cancer treatment specialist	Name of Establishment	Region/Country
A	Surgery	☐$_1$Yes ☐$_2$No	First session: |__|__|/|__|__|/|__|__|			
			Last session: |__|__|/|__|__|/|__|__|			
B	Chemotherapy	☐$_1$Yes ☐$_2$No	First session: |__|__|/|__|__|/|__|__|			
			Last session: |__|__|/|__|__|/|__|__|			
C	Radiotherapy	☐$_1$Yes ☐$_2$No	First session: |__|__|/|__|__|/|__|__|			
			Last session: |__|__|/|__|__|/|__|__|			
D	Other (hormone therapy, alternative medicine etc):		First session: |__|__|/|__|__|/|__|__|			
			Last session: |__|__|/|__|__|/|__|__|			
E	Other (hormone therapy, alternative medicine etc):		First session: |__|__|/|__|__|/|__|__|			
			Last session: |__|__|/|__|__|/|__|__|			

Supplementary materials

F	Other (hormone therapy, , alternative medicine etc):	First session: |__|__|/|__|__|/|__|__|			
		Last session: |__|__|/|__|__|/|__|__|			
G	Other (hormone therapy, alternative medicine etc):	First session: |__|__|/|__|__|/|__|__|			
		Last session: |__|__|/|__|__|/|__|__|			
H	Other (hormone therapy, alternative medicine etc):	First session: |__|__|/|__|__|/|__|__|			
		Last session: |__|__|/|__|__|/|__|__|			

8. Any comments concerning the treatment?

………

………

………

………

Supplementary materials

VIII. Diagnosis and treatment location

1. Was there any part of your diagnosis tests that required you to physically travel outside of Saint Lucia (e.g. MRI or PET Scan)

☐1 Yes ☐2 No

2. Please list the country(ies) travelled to, and the test performed there.

City, Country		Test(s)
A		
B		
C		
D		

Supplementary materials

3. What was(were) the factor(s) that influenced your choice of location for diagnosis? (several responses possible)

Diagnosis Country A ………………………………………………

Personal reasons:	
☐ Attracted by the price of the exam	☐ I don't know
☐ Personal preference for that location	☐ I don't know
☐ Recommendation<br>By who:<br>1- ……………………………………………….<br>2- ……………………………………………….	☐ I don't know
**Practical reasons:**	
☐ Referral by health care provider	☐ I don't know
☐ Location of specific lab/hospital	☐ I don't know
☐ Close location to family/close friend(s)	☐ I don't know
☐ The diagnostic exam was not available in Saint Lucia<br>Specify options which were unavailable locally at the time:<br>1- ……………………………………………….<br>2- ……………………………………………….<br>3- ……………………………………………….	☐ I don't know

☐ Other factor(s) (specify below):

……

……

……

Supplementary materials

Diagnosis Country B ………………………………………………

**Personal reasons:**	
☐ Attracted by the price of the exam	☐ I don't know
☐ Personal preference for that location	☐ I don't know
☐ Recommendation<br>By who:<br>1- ………………………………………………….<br>2- ………………………………………………….	☐ I don't know
**Practical reasons:**	
☐ Referral by health care provider	☐ I don't know
☐ Location of specific lab/hospital	☐ I don't know
☐ Close location to family/close friend(s)	☐ I don't know
☐ The diagnostic exam was not available in Saint Lucia<br>Specify options which were unavailable locally at the time:<br>1- ………………………………………………….<br>2- ………………………………………………….<br>3- ………………………………………………….	☐ I don't know

☐ Other factor(s) (specify below):

……

……

……

Supplementary materials

Diagnosis Country C………………………………………………

**Personal reasons:**	
☐ Attracted by the price of the exam	☐ I don't know
☐ Personal preference for that location	☐ I don't know
☐ Recommendation<br>By who:<br>1- ………………………………………………….<br>2- ………………………………………………….	☐ I don't know
**Practical reasons:**	
☐ Referral by health care provider	☐ I don't know
☐ Location of specific lab/hospital	☐ I don't know
☐ Close location to family/close friend(s)	☐ I don't know
☐ The diagnostic exam was not available in Saint Lucia<br>Specify options which were unavailable locally at the time:<br>1- ………………………………………………….<br>2- ………………………………………………….<br>3- ………………………………………………….	☐ I don't know

☐ Other factor(s) (specify below):

………

………

………

Supplementary materials

4. Was there any part of your cancer treatment that required you to physically travel outside of Saint Lucia (e.g. Radiotherapy etc.)

☐1 Yes, partially

☐2 Yes, I did all my treatment abroad

☐3 No, I did all my treatment in Saint Lucia

....

5. Please list the country(ies) travelled to, the treatment that was administered and the Frequency of your travels to those countries.

Note to interviewer:

- Remind the patient that we are interested in the countries they went to where a treatment was administered and not just a consultation.
- If the frequency of visits to a country is greater than once per month, try to specify the exact number in the column “visits per month”. For visits which were less frequent full out one of the columns in the section “<once every”

<table>
<tr><th colspan="2" rowspan="3">Country</th><th rowspan="3">Treatment</th><th colspan="6">Frequency of travels</th></tr>
<tr><th rowspan="2">once</th><th colspan="2"><once every</th><th rowspan="2">Remained for the entire duration of treatment</th><th rowspan="2">I don’t recall</th><th rowspan="2">Visits per month</th></tr>
<tr><th>1-2 Months</th><th>2-6 Months</th></tr>
<tr><td rowspan="3">A</td><td rowspan="3"></td><td></td><td>☐1</td><td>☐2</td><td>☐3</td><td>☐4</td><td>☐5</td><td>|__|__|</td></tr>
<tr><td></td><td>☐1</td><td>☐2</td><td>☐3</td><td>☐4</td><td>☐5</td><td>|__|__|</td></tr>
<tr><td></td><td>☐1</td><td>☐2</td><td>☐3</td><td>☐4</td><td>☐5</td><td>|__|__|</td></tr>
<tr><td rowspan="3">B</td><td rowspan="3"></td><td></td><td>☐1</td><td>☐2</td><td>☐3</td><td>☐4</td><td>☐5</td><td>|__|__|</td></tr>
<tr><td></td><td>☐1</td><td>☐2</td><td>☐3</td><td>☐4</td><td>☐5</td><td>|__|__|</td></tr>
<tr><td></td><td>☐1</td><td>☐2</td><td>☐3</td><td>☐4</td><td>☐5</td><td>|__|__|</td></tr>
<tr><td rowspan="3">C</td><td rowspan="3"></td><td></td><td>☐1</td><td>☐2</td><td>☐3</td><td>☐4</td><td>☐5</td><td>|__|__|</td></tr>
<tr><td></td><td>☐1</td><td>☐2</td><td>☐3</td><td>☐4</td><td>☐5</td><td>|__|__|</td></tr>
<tr><td></td><td>☐1</td><td>☐2</td><td>☐3</td><td>☐4</td><td>☐5</td><td>|__|__|</td></tr>
<tr><td rowspan="3">D</td><td rowspan="3"></td><td></td><td>☐1</td><td>☐2</td><td>☐3</td><td>☐4</td><td>☐5</td><td>|__|__|</td></tr>
<tr><td></td><td>☐1</td><td>☐2</td><td>☐3</td><td>☐4</td><td>☐5</td><td>|__|__|</td></tr>
<tr><td></td><td>☐1</td><td>☐2</td><td>☐3</td><td>☐4</td><td>☐5</td><td>|__|__|</td></tr>
</table>

Supplementary materials

6. What was(were) the factor(s) that influenced your choice of location for treatment? (several responses possible)

Treatment Country A ……………………………………….

**Personal reasons:**	
☐ Attracted by the price of the treatment	☐ I don't know
☐ Personal preference for that location	☐ I don't know
☐ Recommendation<br>By who:<br>1- …………………………………………………….<br>2- …………………………………………………….	☐ I don't know
**Practical reasons:**	
☐ Referral by health care provider	☐ I don't know
☐ Location of specific clinician	☐ I don't know
☐ Close location to family/close friend(s)	☐ I don't know
☐ The treatment option was not available in Saint Lucia<br>Specify options which were unavailable locally at the time:<br>1- …………………………………………………….<br>2- …………………………………………………….<br>3- …………………………………………………….	☐ I don't know

☐ Other factor(s) (specify below):

………

………

………

Supplementary materials

Treatment Country B ……………………………………….

**Personal reasons:**	
☐ Attracted by the price of the treatment	☐ I don't know
☐ Personal preference for that location	☐ I don't know
☐ Recommendation<br>By who:<br>1- ………………………………………………….<br>2- ………………………………………………….	☐ I don't know
**Practical reasons:**	
☐ Referral by health care provider	☐ I don't know
☐ Location of specific clinician	☐ I don't know
☐ Close location to family/close friend(s)	☐ I don't know
☐The treatment option was not available in Saint Lucia<br>Specify options which were unavailable locally at the time:<br>1- ………………………………………………….<br>2- ………………………………………………….<br>3- ………………………………………………….	☐ I don't know

☐ Other factor(s) (specify below):

………

……..

………

Supplementary materials

Treatment Country C ………………………………………

**Personal reasons:**	
☐ Attracted by the price of the treatment	☐ I don't know
☐ Personal preference for that location	☐ I don't know
☐ Recommendation<br>By who:<br>1- ………………………………………………….<br>2- ………………………………………………….	☐ I don't know
**Practical reasons:**	
☐ Referral by health care provider	☐ I don't know
☐ Location of specific clinician	☐ I don't know
☐ Close location to family/close friend(s)	☐ I don't know
☐ The treatment option was not available in Saint Lucia<br>Specify options which were unavailable locally at the time:<br>1- ………………………………………………….<br>2- ………………………………………………….<br>3- ………………………………………………….	☐ I don't know

☐ Other factor(s) (specify below):

……

……

……

Supplementary materials

7. Do you have family members/close friends living in one of the following countries?

Note to interviewer: Explain where possible to patient, that the countries listed are locations most connected with our health system and where the most exchanges happen habitually for cancer. In addition, specify that "close friend" designates a person outside of their biological family with who could be contacted to assist with housing and logistics for care (being driven to treatment location etc.)

Country	Type of relationship		No one	Don't know
Antigua	☐ Family	☐ Close friends	☐	☐ I don't know
Barbados	☐ Family	☐ Close friends	☐	☐ I don't know
Canada	☐ Family	☐ Close friends	☐	☐ I don't know
Cuba	☐ Family	☐ Close friends	☐	☐ I don't know
Guadeloupe	☐ Family	☐ Close friends	☐	☐ I don't know
Guyana	☐ Family	☐ Close friends	☐	☐ I don't know
Jamaica	☐ Family	☐ Close friends	☐	☐ I don't know
Martinique	☐ Family	☐ Close friends	☐	☐ I don't know
United Kingdom	☐ Family	☐ Close friends	☐	☐ I don't know
The United States of America (USA)	☐ Family	☐ Close friends	☐	☐ I don't know
Trinidad & Tobago	☐ Family	☐ Close friends	☐	☐ I don't know

Supplementary materials

IX. Psycho-social and supportive care

1. Were you asked about your social and home circumstance after your diagnosis and/or during treatment?

   □1 Yes □2 No □3 I don't know

   ……

   ……

   ……

2. Could you describe your experience in coming up with funds to pay for your tests and treatment?

……

……

……

……

……

……

……

……

3. During your treatment, did you know who to contact if you had any questions about your diagnosis or treatment? (e.g. specialist, nurse, care coordinator, cancer patient association)

   □1 Yes □2 No

   ……

   ……

   ……

Supplementary materials

4. Was contact information given in case you had problems or concerns?

☐1 Yes ☐2 No

5. What means were provided to you or your caregiver(s) to contact your Health care provider?

☐ Professional/work line
☐ Personal telephone line
☐ Text messages/SMS
☐ WhatsApp
☐ Email
☐ Social media
☐ Other: ……………………………

………

………

6. Did you feel that you received adequate information about the various care and support services available? Including counselling, physiotherapy, dieting etc.

☐1 Yes ☐2 No ☐3 I don't know

………

………

7. Were you **recommended** or **referred** to any supportive care professional during the course of your journey?

☐1 Yes ☐2 No

8. Did you **have resort** to any supportive care professional? If yes please list them and the point in your journey you did so.

Note to interviewer: Place a tick (√) over the period that corresponds.

Professional	Period during journey
☐ Psychologist	…. Diagnosis …… …… ……. First treatment …… …… …..Follow up ….
☐ Dietician/Nutritionist	…. Diagnosis …… …… ……. First treatment …… …… …..Follow up ….
☐ Exercise coach	…. Diagnosis …… …… ……. First treatment …… …… …..Follow up ….
☐ Physiotherapist	…. Diagnosis …… …… ……. First treatment …… …… …..Follow up ….
☐ Patient navigator	…. Diagnosis …… …… ……. First treatment …… …… …..Follow up ….
☐ Naturopathic professional	…. Diagnosis …… …… ……. First treatment …… …… …..Follow up ….
☐ Other …………………..	…. Diagnosis …… …… ……. First treatment …… …… …..Follow up ….

Supplementary materials

X. Palliation and symptom control

1. Have you been referred to other services for symptom control or support?

   □1 Yes □2 No □3 I don't know

……

……

……

2. Was a referral to palliative care services discussed and available?

   □1 Yes □2 No □3 I don't know

3. Was this referral actioned?

   □1 Yes □2 No

4. Who discussed this referral with you?

……

5. At what point in the care journey was this referral discussed?

……

……

……

……

Supplementary materials

XI. Post-treatment follow up

Note to interviewer: Remind the patient the definition of end of initial active cancer treatment: "shows at this date no visible clinical or biological signs of a recurrence"

1. Have you ended you initial active treatment??

   ☐1 Yes ☐2 No

Note to interviewer: If the patient has ended their initial active cancer treatment. Otherwise, skip to section "**XII – overall perception of experience** "

2. Now that you are done with your initial treatment are you being followed up by a Health care provider?

   ☐1 Yes ☐2 No

3. Please specify the speciality of the health care provider(s) that participate(s) in your follow-up care and the frequency which you visit him/her(them)

Health care provider (HCP)	Every 6 months	Every 12 months	Every 12-24 months	More than 24 months	No specific frequency
☐ General practitioner (GP)	☐1	☐2	☐3	☐4	☐5
☐ Oncologist	☐1	☐2	☐3	☐4	☐5
☐ Specialist doctor (gynaecologist etc.) ………………………………………	☐1	☐2	☐3	☐4	☐5
☐ Naturopathic professional	☐1	☐2	☐3	☐4	☐5
☐ Other allied health professional : ………………………………………	☐1	☐2	☐3	☐4	☐5
☐ Other :………………………………………..	☐1	☐2	☐3	☐4	☐5

Supplementary materials

Additional comments on health care providers:

……

……..

……

4. What does your follow-up care consists of?

Note to interviewer: Prompts the interviewee about information regarding screening test and organisation of follow-up care between them and other HCPs.

……..

……..

……..

……..

……..

5. During these follow up visits with your health care provider, what test/exams(s) do you do?

   Test 1 ……………………………………….

   Test 2 ………………………………………….

   Test 3 ………………………………………….

Supplementary materials

6. Have you been offered any prevention or health promotion interventions from the health care provider(s) who follow you since you have ended your initial active treatment? (i.e. health education, dietary plans, lifestyle coaching)

   ☐1 Yes ☐2 No ☐3 I don't know

……..

……..

XII. Overall perception of experience

1. How would you describe your overall experience of the care received?

□$_1$- Very poor □$_3$-Acceptable □$_5$- Very Good
□$_2$- Poor □$_4$- Good

………

………

2. Did you seek help from Cancer Support Associations (e.g. Faces of cancer etc?)

□$_1$ Yes □$_2$ No

………

………

………

3. Was there anything in particular that made your experience easier?

………

………

………

………

………

Supplementary materials

4. Was there anything in particular that made your experience harder?

………

………

………

………

………

5. Do you have any suggestions to help improve the experience for other people in similar circumstances?

………

………

………

………

………

………

………

………

………

Supplementary materials

XIII. Quality of interview

Note to interviewer: This section must be completed by the interviewer

Interview number …………………………….

End of interview |__|__|h |__|__|

Total duration of interview |__|__|h |__|__| mins

Patients participated in focus group (if several patients)

ID number: |__|__|__| - __ - |__ | __| __|

Rating of general quality of interview (i.e. exchanges with interviewee).

☐1- Poor
☐2- Mediocre
☐3-Good
☐4- Very good
☐5- Excellent

Rating of news responses and novelty of themes emerged from interview (i.e. rate the novelty of the new information provided by the interviewee in the current context of the study).

☐1- No new information and/or Saturation of themes
☐2- Very little new information
☐3-Little new information
☐4- Fairly new amounts of themes emerging
☐5- Very rich in new information

Special remarks on the interview (if any)?

………

……..

………

……..

Supplementary materials
