# Supplementary material for: Difficulties in Accessing Cancer Care in a Small Island State: A Community-Based Pilot Study of Cancer Survivors in Saint Lucia
Source: Int J Environ Res Public Health. 2021 Apr 29;18(9):4770. doi: 10.3390/ijerph18094770 (PMC8124473; doi:10.3390/ijerph18094770)
Supplement: Supplementary file 1 [file ijerph-18-04770-s001.zip › Supplementary files IJERPH_Cancer SLU/Supplementary materials revised 25-04_DCAP.pdf]

Supplementary materials

Title: Difficulties in accessing cancer care in a small island state: a community-based pilot study of cancer survivors in Saint Lucia

Authors: Aviane Auguste, Glenn Jones, Dorothy Phillip, James St. Catherine, Elizabeth Dos Santos, Owen Gabriel, Carlene Radix

Table S1: Brief description of the organisations collaborating on the study design and implementation and communication

Organisation	Type	Description of activities
Faces of Cancer St. Lucia	NGO	Started in 2009 by one of the authors (D.P.), a breast cancer survivor, to assist patients going through their cancer journey. In 2011 FOCS has a membership of over 215 persons from Castries and Dennery. All services and social events offered to survivors of the group are free of charge. Services include education, health fairs emotional and spiritual support, and chemotherapy and post-surgical support. FOCS is currently developing activities beyond cancer advocacy towards involvement in patient navigation and research on cancer risk factors, patient education, health promotion, and access-to-care.
Vaughan Arthur Lewis Institute for research and innovation (VALIRI)	Academic, reasearch	A multi-disciplinary research institute established in 2016 by the Sir Arthur Lewis Community College (Saint Lucia, W.I.). In collaboration with civil society organisations, VALIRI aims to create new knowledge and innovation for the sustainable, economic, and social development of Saint Lucia. VALIRI recruits researchers from the diaspora to serve voluntarily as resource persons for the development of local research projects in areas where their skills are needed. In addition VALIRI also participates in providing an enabling environment and resources where possible. One of the authors (A.A) is a Saint Lucian researcher who contributes to this mechanism.
The Organisation of Eastern Caribbean States (OECS)	inter-governmental	Dedicated to regional integration in the Eastern Caribbean. In 2016, the Health Unit was established and has successfully conducted projects in HIV prevention and surveillance, management of diabetes and reinforcement of the public health response during natural disasters. The OECS has also been charged with developing population-based cancer surveillance in the OECS member states.

NGO: Non-governmental organisation

Table S2: Correlation between education level and the variables hot water at home and private medical insurance among DCAP study participants

Variable	Education level						*p* value
	Primary		Secondary		Tertiary		
	n=16	col%	n=15	col %	n=17	col %	
**Private medical insurance**							0.016
Yes	3	18.7	3	20.0	11	61.1	
No	13	81.3	12	80.0	7	38.9	
**Hot water at home**							0.083
Solar	1	6.7	3	21.4	8	47.1	
Electric	3	20.0	3	21.4	4	23.5	
No	11	73.3	8	57.1	5	29.4	
Missing	1		1		1		

Saint Lucia (West Indies), 2019-2020

p: Exact Fisher test

Table S3: Definition of certain variables collected during interviews

Variable name	Definition
Cancer survivor	A person having been diagnosed with cancer and still alive
Education level	Highest level of education that was completed
Private medical insurance	Being covered by a private health insurance policy at the moment of the interview regardless of the person who pays the policy.
Hot water at home	Availability of hot running water through a heating system in their primary place of residence.
Diagnosis announcement rating	Clarity and adequacy of information conveyed during the announcement of cancer diagnosis were ascertained using a Likert scale with 5 levels of satisfaction: very poor, poor, fair, good and very good.
Adequate information on care support services	Patient perceiving sufficient information on the services in order to make an informed decision on utilising those services.
Recommended or referred to supportive care services	A patient was formally recommended as a part of their treatment plan to consult supportive care professional.
Accessed supportive care services	A patient accessed one of the following professionals at least once between first symptoms and the time of the interview: psychologist, counsellor, nutritionist/dietician, exercise coach, physiotherapist, patient navigator and naturopathic professional
Health promotion intervention	Actions enabling patients to increase control over, and to improve their health in general. Interventions include but are not limited to: lifestyle advice/coaching, health education
Asked about social and home circumstances	The patient being questioned by HCPs on their personal situation relating to family life and financial/material welfare after diagnosis and/or during treatment.
Opportunity to discuss with others	The patient perceived that they were accorded sufficient time to evaluate the decision to accept the prescribed treatment by soliciting advice from persons apart from the prescribing HCP (including family and friends).
Knowing who to contact for information about care	The patient perceived a feeling of certainty when professional medical advice was desired pertaining to their cancer care, including questions about diagnosis or treatment.
HCP contact information was provided	Refers to patient receiving information to contact their HCP in case they had problems or concerns (regardless of the contact medium and the person providing the information)
Sources of funding	Main sources of funds to pay for tests and treatment from an open-ended question.
Overall care experience	A summary of the clinical and non-aspects of participants' journey. This was ascertained using a Likert scale with 5 levels of satisfaction: very poor, poor, fair, good and very good.
Follow-up care	Programmed medical consultations with a professional in the intention of monitoring the status of their cancer after active treatment
Receives health promotion intervention	Offered prevention or health promotion interventions after active treatment from their provider of follow-up care. Interventions include; health education, dietary plans, lifestyle coaching

Communication of study

We did a virtual presentation intended for the local cancer community on world Cancer day 2021, and participants provided feedback.

Table S4: Results from anonymous poll conducted during virtual community presentation for world Cancer day 2021 (n=59).

Question	*n*	*%*	*N° of voters*
**Country of residence**			23
Saint Lucia	12	52	
Caribbean	3	13	
North America	4	17	
Europe	3	13	
Asia/Pacific	1	4	
**Role/position**			23
Cancer survivor	1	4	
Caregiver of cancer survivor	3	13	
Researcher (Ph.D DrPH, other)	3	13	
Physician/Clincian	6	26	
Public health administrator	0	0	
Student	4	17	
Other	6	26	
**Expectations in terms of new information were met**			27
Very low/low	0	0	
Fair	8	30	
High	15	56	
Very high	4	15	
**Desire to participate in similar studies in the future**			27
Yes	22	81	
No	0	0	
Maybe	4	15	
I don't know	1	1	
**Area of priority for future research**			
Cancer risk factors	7	26	
Health services research	13	48	
Not a resident of Saint Lucia	7	26	
**Desire for more private sector funding for health research**			32
Yes	25	78	
No	0	0	
Maybe	3	9	
I don't know	0	0	
Not a resident of Saint Lucia	4	13	

Care pathways

Table S5: Symptoms and reactions to symptoms during self-appraisal interval from survivors discovered their cancer through bodily changes (n=44).

	n	%
**Most common symptoms**		
Lump (anywhere)	19	43.2
Pain (anywhere)	13	29.6
Weight loss	8	18.2
Problems urinating	8	18.2
Bleeding (anywhere)	7	15.9
**Reaction to first symptoms**		
I contacted a HCP	28	66.7
I monitored the symptoms passively	6	14.3
I ignored the symptoms	5	11.9
Brought body changes to family's attention	2	4.8
Self-medicated	1	2.4
Missing	2	
**Reason to consult a HCP**		
Symptoms became very apparent	20	47.6
Encouraged by close friends/family to see HCP	10	23.8
Had a gut feeling something was wrong	8	19.1
Felt overdue for a clinical check-up	2	4.8
Did research on symptoms	1	2.4
Awareness of family history	1	2.4
Missing	2	

Saint Lucia (West Indies), 2019-2020

Table S6: Summary of HCP consultations leading to diagnosis and/or treatment

N° of HCP			Speciality of HCP[†]								Action taken during consultation					
			General practitioner		Specialist		Surgeon		Oncologist		Test		Treatment		Referral	
	n	%[a]	n	% [b]	n	% [b]	n	% [b]	n	% [b]	n	% [b]	n	% [b]	n	% [b]
1	50	27.5	26	52.0	11	22.0	5	10.0	1	2.0	30	68.2	11	26.8	22	50.0
2	49	26.9	10	20.4	18	36.7	9	18.4	8	16.3	26	63.4	13	34.2	19	47.5
3	40	22.0	3	7.7	15	38.5	11	28.2	9	23.1	23	71.9	9	30.0	11	42.3
4	23	12.6	0	0.0	7	30.4	6	26.1	9	39.1	12	63.2	4	26.7	11	55.0
5	13	7.1	1	7.7	5	38.5	2	15.4	4	30.8	8	88.9	5	62.5	5	45.5
6	6	3.3	0	0.0	2	33.3	1	16.7	3	50.0	1	25.0	1	25.0	4	80.0
7	1	0.5	0	0.0	0	0.0	0	0.0	1	100.0	1	100.0	0	0.0	0	0.0
Total	182	100.0	40	22.1	58	32.0	34	18.8	35	19.3	102	67.5	43	31.4	73	49.3

Saint Lucia (West Indies), 2019-2020
a: column percentage, divided by the total number of consultations
b: row percentage, divided by the persons concerned by given number the HCPs
†: Other specialities recorded: Accident & Emergency, Nurse, Primary care (unspecified), Radiologist
Missing data: N° of HCP =1, test=32, treatment=46, referral=35

Table S7: Summary statistics for time to issuance of diagnostic test results by country of exam (weeks)

Type	Saint Lucia			Abroad		
	n	Mean (SD)	Range	n	Mean (SD)	Range
Overall	77	1.9 (3.2)	(0.1-16.0)	43	0.8 (0.8)	(0.1-2.0)
Histology/Cytology	27	4.0 (3.9)	(0.1-16.0)	10	1.2 (0.7)	(0.1-2.0)
Radiology/Imaging	32	0.7 (2.5)	(0.1-14.0)	26	0.8 (0.8)	(0.1-2.0)
Medical biochemisty	18	0.8 (1.2)	(0.1-5.0)	7	0.5 (0.4)	(0.1-1.0)

Saint Lucia (West Indies), 2019-2020
SD: Standard deviation

Table S8: Treatment details from participants included in the study

	n	%
**Country of treatment**		
Abroad, partially	11	26.8
Abroad, full	12	29.3
Saint Lucia only	18	43.9
Missing/No treatment	9	
**Treatment type**		
Surgery	35	83.3
Chemotherapy	30	71.4
Radiotherapy	12	28.6
Hormone therapy	20	45.5
Natural/alternative remedies[a]	5	11.6
Accessed supportive care services[b]	13	33.3
**Post active treatment**		
Regular follow-up care	29	87.9
Receives health promotion intervention[c]	9	37.5

Saint Lucia (West Indies), 2019-2020
a: Papaya leaf tea, soursop leaf tea (leaves, bark and root), tree of life tea
b: Psychologist, counsellor, nutritionist/dietician, exercise coach, physiotherapist, patient navigator and naturopathic professional.

Cancer care abroad

We recorded information from 24 destinations for treatment (23 survivors) and 31 for diagnostic tests (28 survivors). A survivor had the possibility of reporting multiple destinations. Hence, the frequency can exceed the total number of patients who travelled for care.

Table S9: Destinations recorded for medical travels by purpose (test or treatment)

	Diagnostic test[a]		Treatment[b]	
	n	%	n	%
**Latin America & Caribbean**				
Antigua	0	0.0	1	4.2
Barbados	1	3.2	2	8.3
Columbia	1	3.2	1	4.2
Cuba	2	6.5	2	8.3
Guyana	1	3.2	3	12.5
Margarita	1	3.2	1	4.2
Martinique	12	38.7	3	12.5
Saint Thomas	1	3.2	0	0.0
Trinidad	1	3.2	1	4.2
**North America**				
Canada	1	3.2	1	4.2
USA	7	22.6	7	29.2
**Europe**				
UK	2	6.3	1	4.2
**Asia**				
India	1	3.2	1	4.2

Saint Lucia (West Indies), 2019-2020

Margarita: An island which is a part of Venezuela

a: 3 patients travelled to 2 countries and 1 patient travelled to 3 countries for a diagnostic test

b: 1 person travelled to 2 countries for treatment
